# ECG-based prediction algorithm for imminent malignant ventricular arrhythmias using decision tree

**DOI:** 10.1371/journal.pone.0231635

**Published:** 2020-05-14

**Authors:** Satria Mandala, Tham Cai Di, Mohd Shahrizal Sunar, Adiwijaya

**Affiliations:** 1 Human Centric (HUMIC) Engineering, Telkom University, Bandung, Indonesia; 2 School of Computing, Telkom University, Bandung, Indonesia; 3 Media and Game Innovative Centre of Excellence, Institute of Human Centered Engineering, Universiti Teknologi Malaysia, Johor Bahru, Johor, Malaysia; 4 School of Computing, Faculty of Engineering, Universiti Teknologi Malaysia, Johor Bahru, Johor, Malaysia; Universita degli Studi di Catania, ITALY

## Abstract

Spontaneous prediction of malignant ventricular arrhythmia (MVA) is useful to avoid delay in rescue operations. Recently, researchers have developed several algorithms to predict MVA using various features derived from electrocardiogram (ECG). However, there are several unresolved issues regarding MVA prediction such as the effect of number of ECG features on a prediction remaining unclear, possibility that an alert for occurring MVA may arrive very late and uncertainty in the performance of the algorithm predicting MVA minutes before onset. To overcome the aforementioned problems, this research conducts an in-depth study on the number and types of ECG features that are implemented in a decision tree classifier. In addition, this research also investigates an algorithm’s execution time before the occurrence of MVA to minimize delays in warnings for MVA. Lastly, this research aims to study both the sensitivity and specificity of an algorithm to reveal the performance of MVA prediction algorithms from time to time. To strengthen the results of analysis, several classifiers such as support vector machine and naive Bayes are also examined for the purpose of comparison study. There are three phases required to achieve the objectives. The first phase is literature review on existing relevant studies. The second phase deals with design and development of four modules for predicting MVA. Rigorous experiments are performed in the feature selection and classification modules. The results show that eight ECG features with decision tree classifier achieved good prediction performance in terms of execution time and sensitivity. In addition, the results show that the highest percentage for sensitivity and specificity is 95% and 90% respectively, in the fourth 5-minute interval (15.1 minutes–20 minutes) that preceded the onset of an arrhythmia event. Such results imply that the fourth 5-minute interval would be the best time to perform prediction.

## Introduction

Patients with MVA, either ventricular tachycardia (VT) or ventricular fibrillation (VF), always have a risk of sudden cardiac death (SCD). MVA is abnormal heart rate rhythms that occur on the bottom chambers of the heart. Heart rhythm abnormalities are caused by the heart ventricles beating faster than normal. VT may progress to VF. Sustained VT/VF that lasts longer than 30 seconds in duration is malignant, because hemodynamic compromise may happen during the time and could lead to SCD [1].

**Fig 1:**
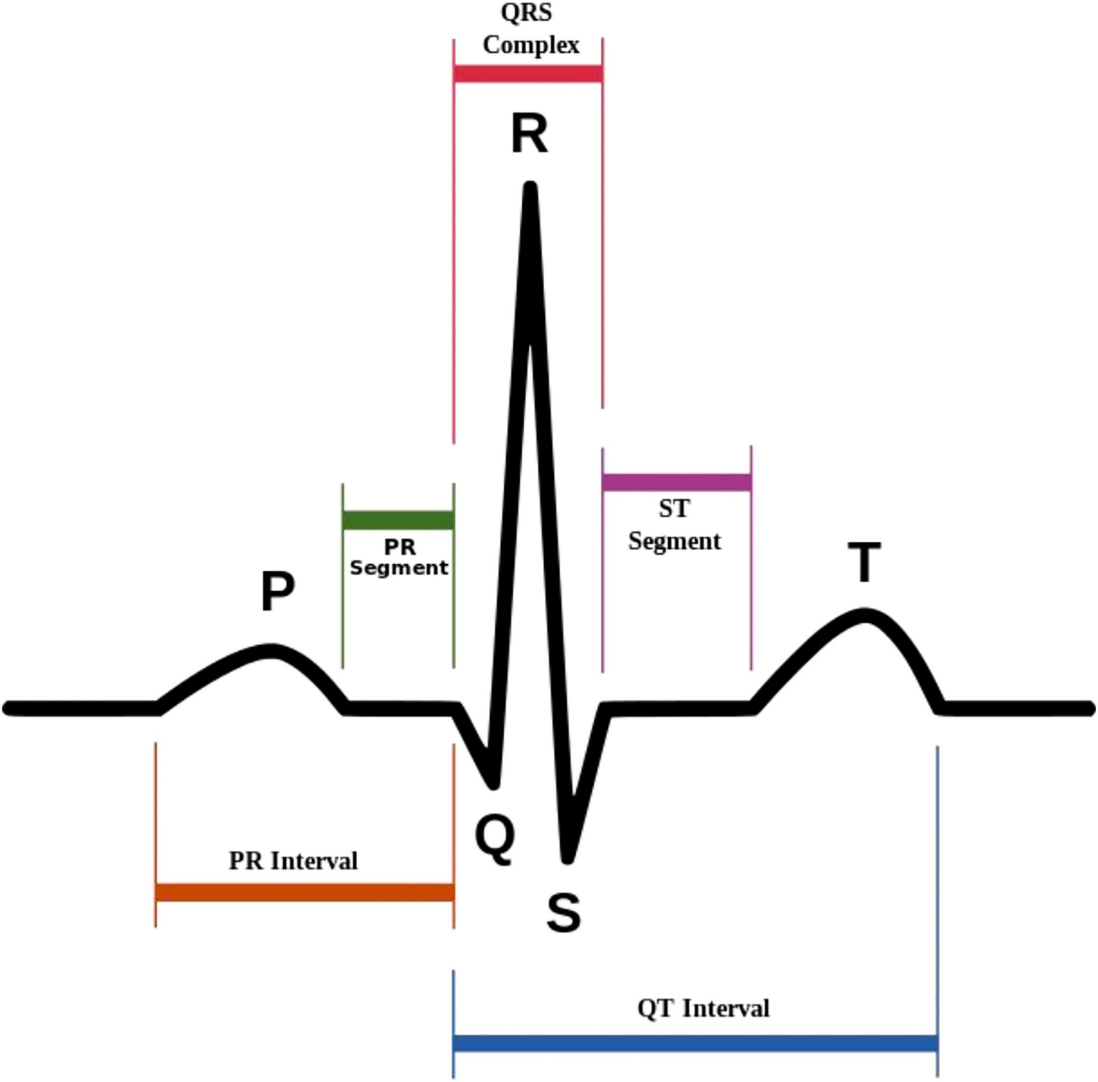
Components of an ECG beat, source: [9].

For detection of the onset of MVA, ECG signal analysis is the most effective method [2]. Fig 1 illustrates the components of an ECG beat. Numerous features derived from intervals, amplitudes and waveform morphology of ECG components are useful to detect underlying VT/VF. For example, mean of RR intervals (mRR), QRS duration (QRSd), mean of QT intervals, and T-wave alternans could be associated with increased risk of MVA [3, 4, 5, 6, 7, 8]. The features may prolong, reduce or precede VT/VF.

ECG is not only useful for detection but also prediction of MVA. Prediction is vital to provide a warning for those who are at risk of VT/VF. Even with a few minutes advance notice of an arrhythmic event, people can do to protect themselves from VT/VF. A notice is helpful because MVA could be treated immediately with medication, electric cardioversion and/or cardiopulmonary resuscitation [10].

In the past, ECG-based long-term risk prediction for the onset of MVA has been the main concern of most researchers [11, 4, 6]. The prediction is to determine whether a patient is at risk for VT/VF over a few months or years. For instance, patient data from years 2007 to 2009 are used to examine the ability of ECG features in predicting the recurrence of VT/VF [6]. Using these features, researchers are able to establish approximate risk stratification, which categorizes patients as belonging to either high or low VT/VF risk group. According to Ragupathi et al. [12], patients with high risk are advised to have an implantable cardioverter defibrillator (ICD). The ICD can give electrical pulses or shocks immediately to get the heart rhythm back to normal when VT/VF occurs.

However, in recent years, researchers have also been interested in the relationship between ECG features and imminent VT/VF, as well as the development of algorithms that can predict an upcoming arrhythmic event [13, 7, 2, 14]. In this study, imminent VT/VF refers to the initiation of VT/VF after a few minutes or hours as stated in previous researches [15, 2, 13]. Researchers were interested in predicting imminent arrhythmia to prevent delays in providing medical assistance to primarily non-ICD patients or people who have no prior history of VT/VF. Furthermore, VT/VF cases are no longer rare, and there are many instances in which VT/VF is proven to be the cause of death [16].

With the rapid development of information and communication technology, giving an early warning to prevent delays in notification for VT/VF can be performed using beeps on any telemedicine devices such as smartphones and smartwatches. The use of smartphones is increasingly popular, because the price of the device is getting cheaper and the device itself has good design [17]. In addition, several communication technologies such as bluetooth are used on Colunas et al. [18, 19, 20]; while Lee et al. [21] utilizes WIFI technology to transmit messages.

As studies on the prediction of an imminent VT/VF episode are few, three questions remain unsolved. First, either single or combinatorial features that perform better prediction is unknown. A different number of ECG features has been implemented in earlier studies for the prediction, and reported results are encouraging. According to these results, single features such as heart rate variability (HRV) or QRSd is a highly specific feature for monitoring immediate risks of fatal VT/VF [4, 5]. On the other hand, combinatorial features with at least three ECG features have higher sensitivity than single feature [3, 13, 7, 2]. However, reported results of the studies cannot be directly compared because these studies are based on various ECG data sources and prediction algorithms. Hence, the performance of the single feature and combinatorial features for prediction remains unclear.

Second, the time required for an algorithm to execute a prediction for imminent MVA is also unknown, because it is usually neglected in existing studies [15, 13, 7, 2, 8]. Instead of execution time (exT), the performance of prediction algorithms is evaluated in terms of accuracy, sensitivity (SE) and specificity (SP). Neglect of exT may cause a delay in providing alert for imminent VT/VF. In particular, both longest possible prediction time and best timing to perform the prediction, so far, have been uncertain. The uncertainty is due to different possible prediction times before the onset of imminent VT/VF that are demonstrated in the past studies. The time would be one hour, several minutes or even a few seconds preceding the onset [15, 13, 8, 14]. If possible prediction time is short, self-rescue time of potential patients is also short. Therefore, any delay that further reduces time to rescue patients from VT/VF should be minimized.

Third, the performance of the algorithm in terms of prediction of time is questionable. According to Ebrahimzadeh et al. [15], the performance of ECG-based prediction for SCD has decreased over time. As SCD is always caused by VT/VF, a similar problem may also arise in predicting the arrhythmic event. Besides, a change in mRR starts at least 20 minutes before the onset of VT/VF, and no significant difference could be observed in an earlier time, unless combinatorial features are used [7]. Although 20 minutes will be shortened to several minutes using combinatorial features, such observation indicates there is a certain period that an ECG-based prediction is useless. On the contrary, combinatorial features in the research of Bayasi et al. [2] succeeded in predicting VT/VF up to three hours before its initiation, but if the prediction was always accurate within hours is unknown. Thus, there is a chance that the algorithm will fail to predict minutes before the onset.

In order to answer these three questions, this study aims to propose a fast ECG-based prediction algorithm for imminent VT/VF using a decision tree (DT) while maintaining good sensitivity and specificity. In accordance to the aim of this study, three research objectives are formulated as follows:

1. To identify the optimal number of ECG features that can reduce the exT for predicting imminent VT/VF with low or no reduction in SE and SP.
2. To develop an algorithm for prediction of an arrhythmic event by applying an optimal feature set into a classifier.
3. To evaluate the performance of proposed algorithm in five 5-minute intervals, which are 5 minutes, 10 minutes, 15 minutes, 20 minutes, and 25 minutes before the onset of a what could be fatal arrhythmic event.

## Databases

This study acquired ECG records from two databases namely MIT-BIH Normal Sinus Rhythm Database (NSRDB) and MIT-BIH Malignant Ventricular Ectopy Database (VFDB), which are public data sources from Physionet [22]. There are reference annotation files supplied for the databases to aid users in locating events of interest. VT/VF onset annotations mark only the rough beginnings of VT/VF episodes. The NSRDB includes records of subjects from Boston’s Beth Israel Hospital (now the Beth Israel Deaconess Medical Center) who have no significant arrhythmias, with a sampling rate of 128 Hz. The database has 18 records, each consists of 11,730,944 samples (slightly less than 25.5 hours). The subjects from the database are five men aged 26–45 and 13 women aged 20–50. On the other hand, the VFDB has 22 records with a sampling rate of 250 Hz, and each contains 525,000 samples (35 minutes). Unlike the NSRDB, the VFDB lacks gender and age information from the records. The two databases have a total of 40 records.

However, only 18 of the 40 records were involved in this study, because only nine VFDB records with apparently normal beats have identifiable QRS complexes preceded VT/VF episodes were required in order to find out subtle changes in normal beats. The records indicate that patients seemed like normal, but most were in need of prediction for imminent VT/VF. Other records from VFDB were excluded because of unidentified QRS complexes in most ECG signal beats in the records. According to the annotation files of the VFDB on Physionet, the unidentified QRS complexes are caused by the occurrence of VT, first-degree heart block, ventricular bigeminy, nodal rhythm, or supraventricular tachycardia [22]. Another nine records that represent normal beats are acquired from NSRDB.

Table 1 lists the records used in this study. Before importing into MATLAB, the records with .dat format were converted into the .mat format that fits with MATLAB. The conversion was done using a program from Physionet named wfdb2mat.

**Table 1:** List of records used in this study.

Database	Records
VFDB	420, 421, 422, 423, 425, 426, 427, 605, 612
NSRDB	16265, 16272, 16273, 16420, 16483, 16539, 16773, 16786, 17052

## Methods

The algorithm consists of four modules: ECG preprocessing followed by feature extraction, feature selection, and feature classification.

### ECG preprocessing

The ECG preprocessing module includes two major steps namely ECG filtering and fiducial point detection. ECG filtering involves transformation of raw ECG signals into a comprehensive and noiseless format. Raw data are always noisy, unreliable or comprises much irrelevant and redundant information [13]. On the other hand, fiducial point detection aims to detect fiducial points of ECG, which are the R-peak, onset point of Q-wave (Qon) and offset point of S-wave (Soff). In an ECG signal, R-peaks are the most significant waveform, as illustrated in Fig 1.

This module was developed based on the adaption of MATLAB Pan Tompkins algorithm (PT) by Sedghamiz [23]. PT incorporated several fundamental techniques such as filtration using a bandpass filter, squaring, adaptive thresholding, and windowing. The module kept filters (bandpass filter and derivative filter) and R-peaks detection methods in the original work. Before filtration, a total of 18 ECG records from NSRDB and VFDB were truncated to 35 minutes (maximum length of each record in VFDB) in order to maintain the consistency of the records in length. Then the module replaces outliers in each 35-minute length ECG record with their respective previous value before filtration to maximize the detection rate of fiducial points in the next module. After that, the module partitions each ECG record from either NSRDB or VFDB into thirty-five 1-minute segments. The reason for choosing the one-minute interval is that only VT/VF episodes that sustained longer than 30 seconds were considered malignant. All the segments after the first one-minute segment with sustained VT/VF were excluded from the experiment, because this study only analyzes signal beats from before the onset of VT/VF. Due to different sampling rates, the one-minute segments that originated from NSRDB and VFDB have different sample size, which is 7,680 and 15,000 respectively.

With each detected R-peak, respective Qon and Soff could be identified using simple search windows and zero slope detectors. Using the backward and forward search windows of R-peaks, the local minima before and after each R-peak is the Q and S points, respectively [13]. According to Bayasi et al. [2], the point that has a nearly zero slope and precedes an R-peak is known as the onset point, whereas the point that has a nearly zero slope after the R-peak is determined as the offset point.

### Feature extraction

The feature extraction module aims to extract features from fiducial points of ECG signals. Previous studies have preferred to use techniques that require complex transformation or analysis of extraction modules such as neural network and support vector machine (SVM) [24, 25, 26]. Such techniques usually extract with high accuracy but also increase the overall cost of algorithm [2]. Therefore, in recent years, some studies have tended to use simple extraction methods such as mathematical ECG morphology, which is fast but maintains good accuracy [13, 2]. The mathematical ECG morphology technique computes ECG features based on peaks and onset and offset points.

In this study, the mathematical morphology technique was selected to extract 12 features due to its good tradeoff between speed and accuracy [27, 28]. The first five features were time-domain R-peaks related features in the experiment named mRR, mean of heart rate (mHR), standard deviation of normal-to-normal RR intervals (SDNN), root mean square of the successive differences (RMSSD), and mean of QRS duration (mQRSd). To explore deeper into the change in Q-R-S points that precede the onset of VT/VF, the study included another seven features derived from the mean and standard deviations of intervals and amplitude of Q-R-S points. In addition to the standard deviation of QRS duration (sdQRSd), another six features were mean and standard deviation for the amplitude of Q (mQamp and sdQamp), R (mRamp and sdRamp), and S Points (mSamp and sdSamp). The features were measured in different units; for instance, heart rate was in beats per minute unit, duration features were in second time unit, whereas amplitude features were in millivolt unit.

Eqs 1 to 4 show computation of features that could not be obtained directly from the morphology of ECG. Note that RR interval (RRi) referred to the interval between the R-peak in a beat and the R-peak in the next beat.

1. HR is the number of times the heart beats per minute, and its calculation is as shown in Eq 1 [29].
  $$\begin{array}{c} HR=60/(\sum RR_{i}/fs), \end{array}$$ (1)
  where fs = sampling frequency.
2. mRR is the mean of all RR intervals, which can be calculated using Eq 2 [15].
  $$\begin{array}{c} RR_{mean}=1/n\sum RR_{i}, \end{array}$$ (2)
  where n = total number of beats.
3. RMSSD is the square root of the mean squares of differences between adjacent RR intervals, which can be computed using Eq 3 [15].
  $$\begin{array}{c} RMSSD=\surd 1/n\sum {\left(RR_{i+1}-RR_{i}\right)}^{2}, \end{array}$$ (3)
  where n = total number of beats.
4. SDNN is the standard deviation of all RR intervals, and its calculation is as indicated in Eq 4 [15].
  $$\begin{array}{c} SDNN=\surd 1/n\sum {\left(RR_{i}-RR_{mean}\right)}^{2}, \end{array}$$ (4)
  where n = total number of beats.

### Feature selection

The feature selection module aims to choose the best-describing subset of input features from the original set of ECG features. The module is important because the performance of the proposed prediction algorithm can be strongly affected by the number and relevance of the input features [13]. Without selection, redundant features can increase exT and decrease the general performance of the algorithm [15].

In this study, a MATLAB function, estimates of predictor importance (Imp) and 10-fold cross-validation (CV) were chosen to select the optimal feature set because these methods are suitable for the study that involves a small number of input datasets. Such CV was repeated five times using random number generator (RNG) = 1–5 to ensure the reliability of results. Numbers 1-5 are the seeds to generate random numbers that are repeatable. Every time the generator is initialized using the same seed, the same experiment result will be produced [30]. Imp is a method that considers both interactions and correlations of features [31]. Based on MATLAB manual [30], the Imp is a MATLAB built-in function that is available for DT. The Imp function computes estimates for the DT by summing up changes in the mean squared error (MSE) due to splits in every feature and dividing the sum by the number of branch nodes of the DT. Then the function outputs a sequence of features from the most important to the least important.

On the other hand, the CV is a widely used method for estimating test errors if there is limited input datasets for algorithm evaluation [15]. The k-fold CV is a better choice for time-series data because there is no overlapping between data. The CV is repeated k-times, leaving one different fold for each testing time. In other words, k-1 folds are used for training, and the last fold is used for evaluation or testing. k = 5 or 10 provides good compromise in bias-variance tradeoff [32]. If the number of datasets for the evaluation is lesser, then a smaller number of k is used [15]. There are two variants of the k-fold CV, which are performing feature selection before splitting data into folds (OUT) and performing feature selection k-times inside the CV Loop (IN) [33, 34]. The OUT involves both training and testing sets, whereas the IN only looks at the training set. According to Refaeilzadeh et al. [33], the OUT is a better choice for the feature selection module, even if it demonstrates a larger bias than IN in estimating accuracy, because OUT has a lower bias imbalance. Besides, if the number of input datasets is limited, the IN method could cause a large variance in the performance of feature selection.

### Feature classification

The feature classification module aims to discriminate normal and abnormal ECG that reflects the risk of VT/VF. The DT was selected as classifier because of its fastest worst-case time complexity. The fast worst-case time of the DT could provide more time for a patient to rescue oneself from fatal VT/VF. Moreover, the OUT based 5-fold CV method was chosen for evaluation in the classification module. The number of folds for the CV was less than those in the feature selection module because of a lesser number of available datasets for classification. During classification, the feature sets obtained from the feature selection module are analyzed based on a 5-minute interval, because such interval is recommended for short-term analysis of R-related components [35, 14]. This study compared the DT with naive Bayes (NB) and SVM, which are two different classifiers used in literature published in the last five years. In a MATLAB environment, the functions to train the DT, NB, and SVM classifiers are **fitctree, fitcnb**, and **fitcsvm**, respectively, whereas the function to test is **predict**. The evaluation for the two situations was repeated five times using a function, RNG = 1, 2, 3, 4, 5. The repetition was to achieve a reliable evaluation by averaged resulting values, whereas the RNG was to control the random number generation on MATLAB.

## Results

In this paper, a DT based prediction algorithm with an optimal feature set is proposed. To examine the number of features for generating the optimal feature set (in feature selection module) and for demonstrating better performance of the proposed algorithm if the DT classifier was applied (in feature classification module), the results of the evaluation are collected based on the following situations:

1. Estimates of MSE of different numbers of features with 10-fold CV
2. Different sizes of feature set and DT with 5-fold CV
3. Different classifiers with 5-fold CV and optimal feature set.

In this study, the error-based measures (SE and SP) and exT are selected as performance measures for evaluation. The error-based measures are better choices for an evaluation that involved a comparison of several classifiers. Besides, both SE and SP provide distinction between false positive (FP) and false negative (FN), as shown in Eqs 5 and 6. On the other hand, for the measurement of the exT of the proposed prediction algorithm, the MATLAB **timeit** function, was selected to measure the exT required to run the proposed algorithm.

1. The SE is a proportion of patients with a positive test, where
  $$\begin{array}{c} \text{SE}=\frac{\text{TP}}{\text{TP}+\text{FN}} \end{array}$$ (5)
  and TP is true positive.
2. SP is the proportion of healthy individuals with a negative test, where
  $$\begin{array}{c} \text{SP}=\frac{\text{TN}}{\text{TN}+\text{FP}} \end{array}$$ (6)
  and TN is true negative.

Interpretation of the three performance measures for the evaluation result was different. For SE and SP, the interpretation was that the higher the percentage, the better the SE or SP. For exT, the interpretation was that the shorter or lower exT, the faster the proposed algorithm. In this study, high SE was the top priority performance measure, followed by low exT and high SP. High SE indicates high rate of correct warning for people who are at risk of imminent VT/VF. Low exT means the time required by an algorithm to generate a critical warning is short. High SP represents a low rate of receiving false warning. In other words, SE affects the chance of survival; exT has an impact on the available time to prevent a patient from dying; and SP influences the credibility of the prediction algorithm. Both SE and SP were expressed in percentage, whereas exT was presented in millisecond (ms).

### Selection of optimal feature set

In the first situation, the module implemented MATLAB function Imp to estimate the relative importance of each feature in the prediction of imminent VT/VF. Imp zero represents the smallest possible importance [30]. According to the result of Imp, the features from most important to least important are mQRS, followed by sdSamp, mQamp, mHR, mSamp, mRR, sdQamp, SDNN, RMSSD, sdQRSd, mRamp, and sdRamp. Table 2 shows estimates of Imp for the aforementioned 12 features. As stated in MATLAB manual [30], Imp indicates the relative importance of each feature for prediction; Imp zero represents the smallest possible importance. Hence, four features with Imp zero in the study namely RMSSD, sdQRSd, mRamp, and sdRamp are assumed to have less or no impact on the prediction of onset of VT/VF.

**Table 2:** Estimates of Imp for 12 features.

No.	Features	Imp
1	mQRSd	0.0145
2	sdSamp	0.0060
3	mQamp	0.0041
4	mHR	0.0037
5	mSamp	0.0021
6	mRR	0.0021
7	sdQamp	0.0013
8	SDNN	0.0012
9	RMSSD	0
10	sdQRSd	0
11	mRamp	0
12	sdRamp	0

**Fig 2:**
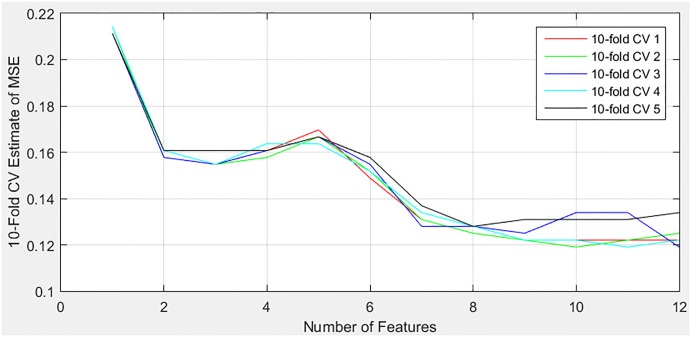
Estimates of MSE of different number of features.

This study further confirmed the assumption by conducting estimations of MSE using the 10-fold CV to find out the number of features needed for prediction of imminent VT/VF. Every estimation was performed by adding one new feature in the sequence. Such trial was repeated five times, and results were collected. Fig 2 illustrates the relationship between the number of features and the estimates of MSE, with the details listed in Table 3. In general, the MSE would be reduced by an increasing number of features and less fluctuated during trials. Table 3 shows the reduction in the average column and fluctuation in the standard deviation (SD) column, the closer the MSE to zero, the higher the rate of correct prediction for impending VT/VF; the higher the SD, the larger the fluctuation of MSE. However, the MSE did not always decrease with every additional feature.

**Table 3:** Estimates of MSE of different number of features.

No. of features	MSE						
	Trial 1	Trial 2	Trial 3	Trial 4	Trial 5	Average	SD
1	0.2113	0.2143	0.2113	0.2143	0.2113	0.2125	0.0016
2	0.1607	0.1577	0.1577	0.1607	0.1607	0.1595	0.0016
3	0.1548	0.1548	0.1548	0.1548	0.1607	0.1560	0.0027
4	0.1607	0.1577	0.1607	0.1637	0.1607	0.1607	0.0021
5	0.1696	0.1667	0.1667	0.1637	0.1667	0.1667	0.0021
6	0.1488	0.1518	0.1548	0.1518	0.1577	0.1530	0.0034
7	0.1310	0.1310	0.1280	0.1339	0.1369	0.1321	0.0034
8	0.1250	0.1250	0.1280	0.1280	0.1280	0.1268	0.0016
9	0.1220	0.1220	0.1250	0.1220	0.1310	0.1244	0.0039
10	0.1220	0.1190	0.1339	0.1220	0.1310	0.1256	0.0065
11	0.1220	0.1220	0.1339	0.1190	0.1310	0.1256	0.0065
12	0.1220	0.1250	0.1190	0.1220	0.1339	0.1244	0.0057

Fig 2 depicts that there is a sharp decrease in the MSE when a couple of features are used. On average, Table 3 shows that the MSE is reduced from 0.2125 to 0.1595. However, the MSE increased if more than three features were used. The MSE even reached the second highest peak of 0.1667 when the number of features was five. Then, the MSE decreased below its lowest value before the first rise and succeeded to achieve the minimum of 0.1244 averagely when nine features were involved. Any additional feature after this had no impact on the reduction of MSE. The result was according to Imp in Table 2. The ninth feature, which is RMSSD, had the least importance. However, trials with nine features were more fluctuated compared with trials with eight features, and their respective SD were 0.0039 and 0.0016. The fluctuation indicates that the former has less consistent MSE, and some trials may have higher MSE than the latter.

Thus, there was another experimental test on the impact of different numbers of features on the proposed algorithm in terms of SE, SP, and exT. Table 4 shows that the DT implemented with eight and nine features have identical SE but slightly different SP, whereas exT of the DT increases as the number of features increases. In this study, high SE is the priority measure, followed by low exT and last is high SP. In this case, the study is better to group eight features as optimal feature set. With the optimal feature set, the exT of the algorithm could reduce 17.95% of the exT in exchange for 0.56% SP reduction when eight features were grouped.

**Table 4:** Average performance of DT with different numbers of features.

Number of features	SE (%)	SP (%)	exT (ms)
8	89.28	79.33	0.64
9	89.28	79.78	0.78

### Performance of decision tree with different sizes of feature set

In the second situation, the study evaluates the proposed algorithm when it applied the DT classifier with different sizes of feature sets, which is the optimal set and the full set in predicting imminent VT/VF. Note that the DT of the optimal set is denoted as oDT, whereas the DT of the full set is referred to as fDT. Table 5 provides a comprehensive comparison of SE, SP, and exT between oDT and fDT at five 5-minute intervals that precede the onset of VT/VF. Performance measures were used to determine which feature set could improve the performance of the proposed algorithm. The oDT showed good tradeoff among the three performance measures. On average, the proposed algorithm with oDT could greatly reduce the exT required (20.50%) and slightly improve SE (0.44%) in exchange for small SP reduction (5.81%). Moreover, the oDT had more consistent performance than the fDT because of its lower SD in all performance measures, indicating that the oDT is less spread out on average.

**Table 5:** Comparison of performance of oDT and fDT.

Minutes before VT/VF	oDT			fDT		
	SE (%)	SP (%)	exT (ms)	SE (%)	SP (%)	exT (ms)
≤ 5	86.67	77.78	0.6419	80.00	88.89	0.8256
≤ 10	88.89	68.89	0.6498	91.11	75.56	0.8138
≤ 15	82.50	80.00	0.6491	85.00	80.00	0.7912
≤ 20	95.00	90.00	0.6416	95.00	90.00	0.7920
≤ 25	93.33	80.00	0.6394	93.33	86.67	0.8305
Average	89.28	79.33	0.6444	88.89	84.22	0.8106
SD	5.05	7.52	0.0048	6.25	6.21	0.0184

**Fig 3:**
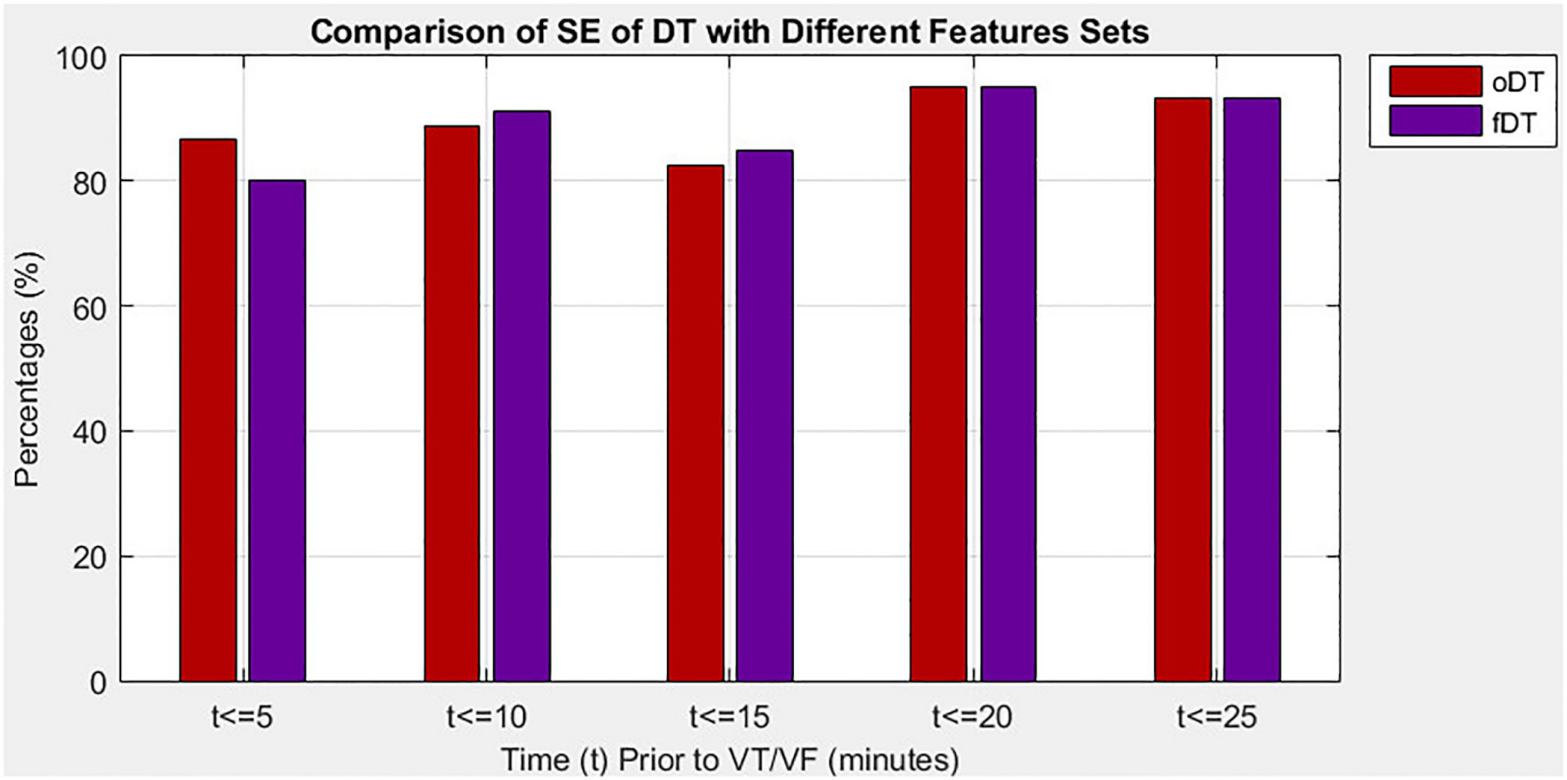
Comparison of SE for DT with different sizes of feature set.

Fig 3 outlines the SE of oDT and fDT in five 5-minute intervals before a VT/VF event, with details listed in Table 5. In the first 5-minute interval, the oDT had higher SE than fDT, 86.7% versus 80%. The percentage indicated that 86.7% of patients was correctly identified using the oDT but only 80% were correctly detected if the fDT was used. However, in the next two 5-minute intervals, which was the 5.1 minutes–15 minutes interval that preceded the event, the oDT demonstrated slightly lower SE than the fDT. During the intervals, at least 11.1% of patients was falsely recognized as normal. The oDT achieved the highest percentage of SE in the fourth 5-minute interval. Moreover, the oDT had the same SE as the fDT starting from the fourth 5-minute interval, implying that the oDT was as sensitive as the fDT 15.1 minutes before VT/VF.

**Fig 4:**
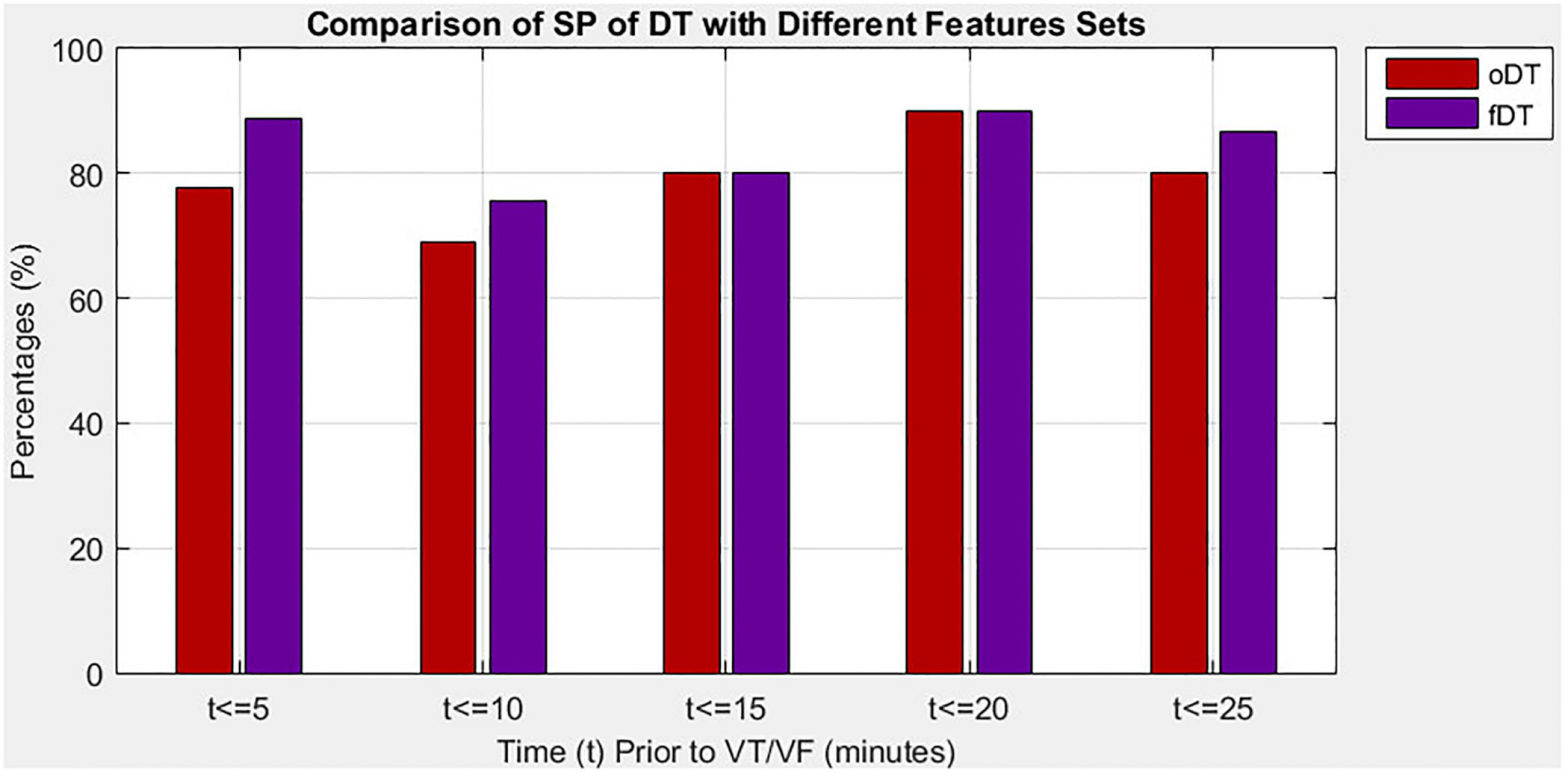
Comparison of SP for DT with different sizes of feature set.

Fig 4 illustrates the SP of the oDT and fDT in five 5-minute intervals prior to the onset of VT/VF, with details listed in Table 5. Overall, the oDT was less specific than the fDT in recognizing individuals without fatal VT/VF. However, the oDT was still considered very specific to such prediction beginning between the third 5-minute interval and afterward because it achieved at least 80% of the SP. In the fourth 5-minute interval, the highest SP of 90% was reported, the higher the percentage, the lower the rate of FP. The percentage of SP indicated that 90% of healthy individuals was correctly recognized as normal using the oDT 15.1 minutes–20 minutes before onset.

**Fig 5:**
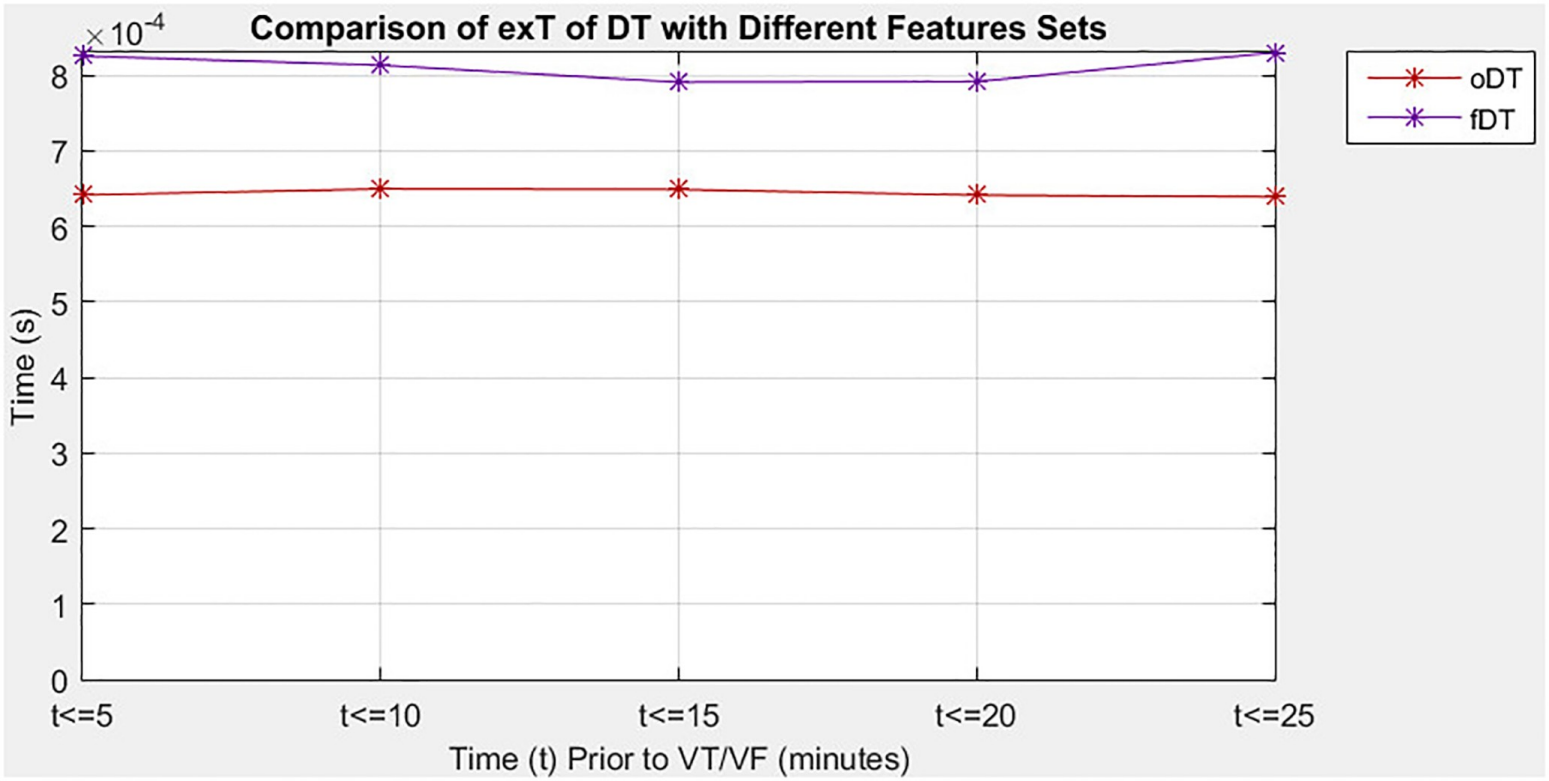
Comparison of exT for DT with different sizes of feature set.

Fig 5 depicts exT of the oDT and fDT to perform prediction in five 5-minute intervals before VT/VF. The oDT required much shorter exT compared to the fDT for all intervals before the arrhythmic event. The oDT needed about 0.64 ms to perform a prediction of imminent VT/VF. On the other hand, the fDT consumed more than 0.79 ms to carry out a classification task for prediction purpose. The result indicated that the number of features had a direct impact on exT.

### Performance of decision tree against other classifiers

In the third situation, the study evaluates the performance of the oDT and two other classifiers, which are NB and SVM, in predicting imminent VT/VF. The performance of the three classifiers was evaluated based on the same optimal features, type of CV as well as performance measures. Table 6 shows a comparison of performance measures among the three classifiers, namely oDT, NB, and SVM. The comparison results revealed that the oDT had better tradeoff in terms of SE, SP, and exT. On average, the oDT had an increase of at least 15.29% in SE compared with NB and SVM. Besides, the oDT reduced 79.03% of exT compared with NB. Although the oDT had the lowest SP among the classifiers, it was still considered specific to the prediction of imminent VT/VF, because its average SP was nearly 80%. Therefore, the oDT was considered as a fast classifier that could yield a good SE gain in exchange for an acceptable SP loss (not more than 13.54%). The performance of the oDT was fairly consistent during the five 5-minute intervals. The consistency could be viewed from the SD values of the oDT. The oDT had the least SD of SE (5.05%), less fluctuated SP compared with SVM (7.52% and 9.14%) and much lesser fluctuation of exT than NB (0.0048 and 0.0597).

**Table 6:** Comparison of performance among DT, NB, and SVM.

Minutes before VT/VF	oDT			NB			SVM		
	SE (%)	SP (%)	exT (ms)	SE (%)	SP (%)	exT (ms)	SE (%)	SP (%)	exT (ms)
≤ 5	86.67	77.78	0.6419	71.11	95.56	3.0353	28.89	100.00	0.5785
≤ 10	88.89	68.89	0.6498	77.78	88.89	3.0548	37.78	84.44	0.5798
≤ 15	82.50	80.00	0.6491	70.00	97.14	3.0387	50.00	94.29	0.5769
≤ 20	95.00	90.00	0.6416	75.00	90.00	3.0575	60.00	80.00	0.5728
≤ 25	93.33	80.00	0.6394	93.33	86.67	3.1782	73.33	100.00	0.5725
Average	89.28	79.33	0.6444	77.44	91.65	3.0729	50.00	91.75	0.5761
SD	5.05	7.52	0.0048	9.41	4.49	0.0597	17.6	9.14	0.0033

**Fig 6:**
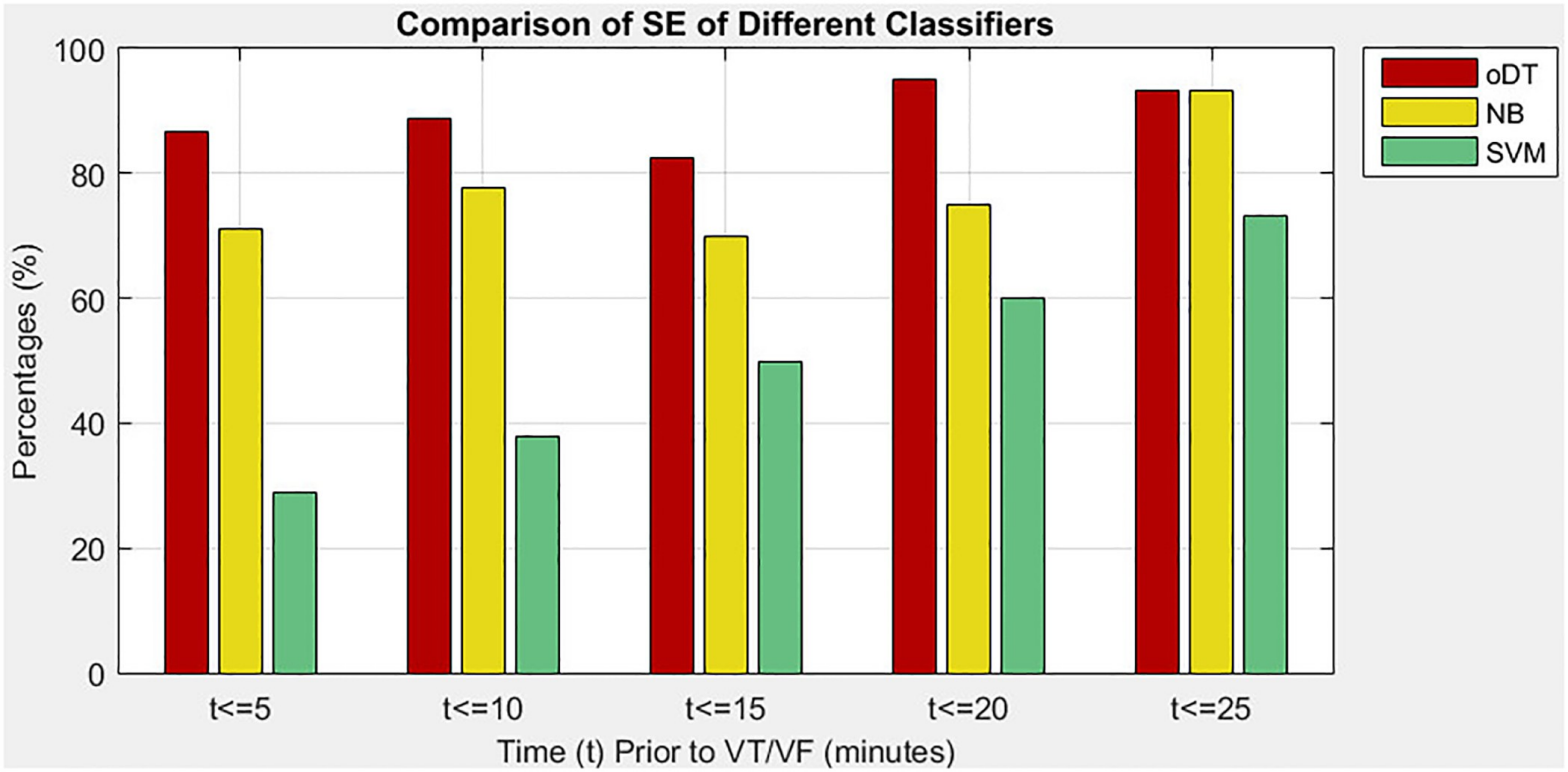
Comparison of SE among oDT, NB and SVM.

Fig 6 shows the SE of three classifiers namely oDT, NB, and SVM in five 5-minute intervals before VT/VF event. Among the classifiers, the oDT maintained its SE superiority in all intervals, which ranged from 82.5% to 95%. The result indicated that at least 82.5% of patients with imminent VT/VF were correctly recognized. On the other hand, the SVM had the lowest SE in such prediction of as low as 28.9% at the first 5 minutes before VT/VF. The low SE implied that the SVM would produce more FN alerts than the other two classifiers. Interestingly, the SE of SVM was increasing gradually with time, so the SVM may be the most sensitive classifier at least half an hour before the initiation of VT/VF.

**Fig 7:**
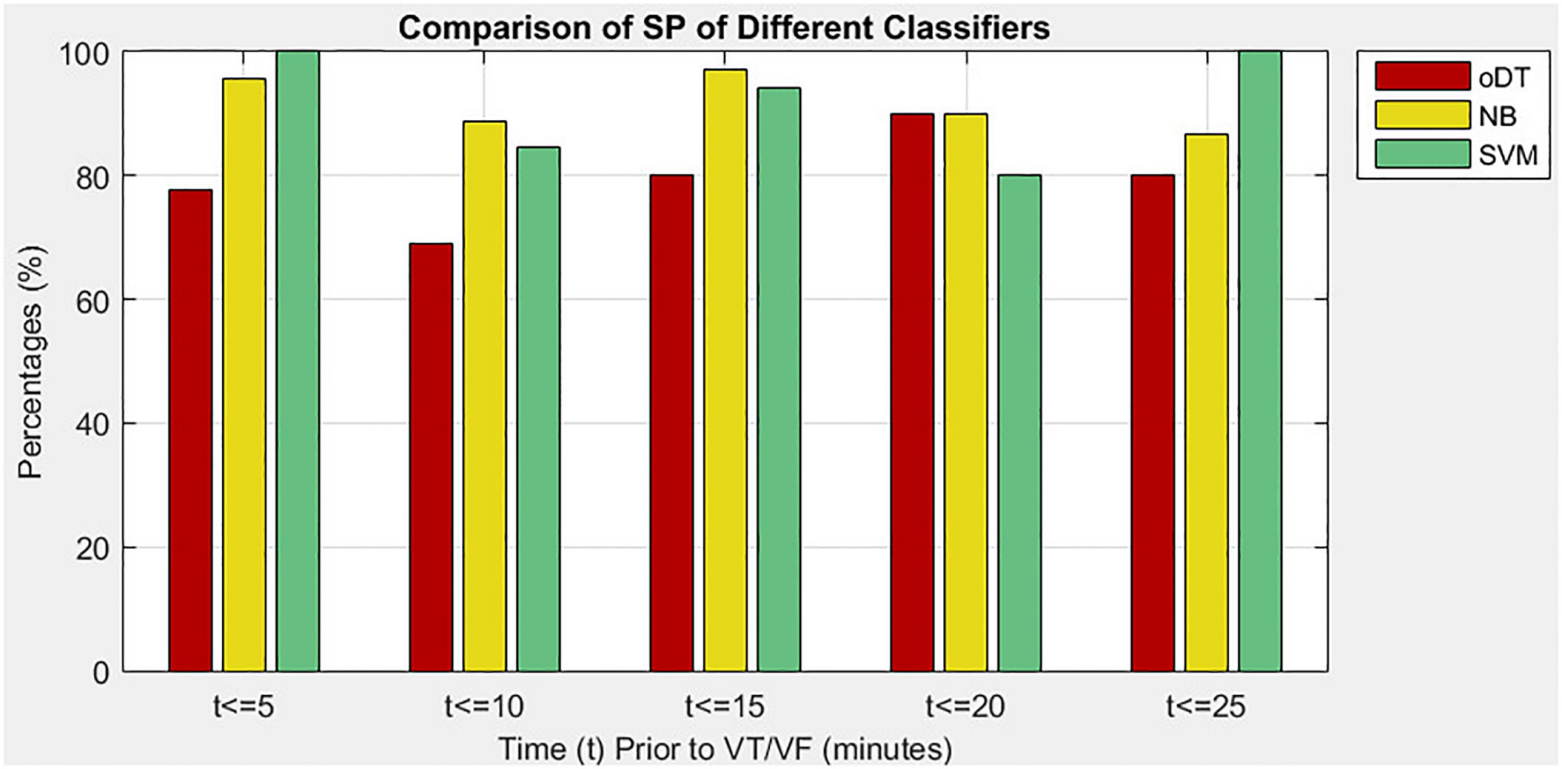
Comparison of SP among oDT, NB and SVM.

Fig 7 exhibits the SP of the three classifiers in five 5-minute intervals that precede the onset of VT/VF. Results revealed that the oDT had a relatively lower SP than the NB and SVM. The oDT even reached a minimum of 68.9% in the second 5-minute interval, which was the 5.1 minutes–10 minutes that preceded the onset of VT/VF. The percentage meant that 31.1% of healthy individuals was incorrectly identified as patient at risk of VT/VF. However, in the fourth interval, the SP of the oDT was as good as the NB and better than SVM. Therefore, the oDT was considered highly specific 15.1 minutes–20 minutes before onset.

**Fig 8:**
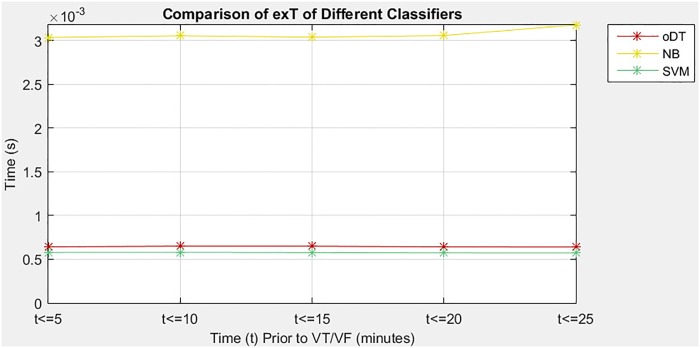
Comparison of exT among oDT, NB and SVM.

Fig 8 illustrates the exT required by the three classifiers to carry out prediction in five 5-minute intervals before VT/VF. Among the classifiers, the DT required the second shortest exT to complete a prediction, which was approximately 0.64 ms. Such result was slightly worse than the SVM but better than the NB that spent at least 3.04 ms to accomplish the same task.

## Discussion

Table 7 shows a list of studies on prediction of imminent VT/VF in the past decade. Most performance results of the studies are either highly sensitive or highly specific (SE/SP ≥ 80%) but not both except for the Riasi et al. study [13]. However, the features in the study are extracted within the last 20 seconds of ECG signals before VT and control records, and such period may be insufficient for a patient to take action.

**Table 7:** List of prediction studies for imminent VT/VF.

Authors	ECG features	Classifiers	Performance	
			SE (%)	SP (%)
Joo et al. [3]	11 HRV features	NN	82.9	71.4
Rozen et al. [5]	HRV	-	50	91.6
Riasi et al. [13]	10 features from QRS	SVM	88	100
Wollmann et al. [7]	3 HRV features	-	94.4	50.6
Bayasi et al. [2]	7 features from intervals of P-QRS	NB	99.83	-

Results in the “Selection of optimal feature set” section revealed that a good prediction algorithm for imminent VT/VF preferred combinatorial features to single feature. The results suggested that eight features derived from QRS complexes could be combined as an informative set for prediction. The eight features were mQRSd, sdSamp, mQamp, mHR, mSamp, mRR, sdQamp, and SDNN. If only one feature was used, the highest MSE was generated, but the MSE dropped obviously when at least two features were involved. The high MSE may imply a high failure rate of a single feature in such prediction. For example, a study by Sachdev et al. [36] showed many single features derived from ECG components such as mHR, mRR, and QT interval failed to predict impending VT/VF. Even though prior studies [6, 7] had succeeded in predicting arrhythmia using a single feature, their reported SE and SP were much lower than studies with combinatorial features [13, 2]. The reported SE and SP in the former was about 50% compared to above 85% in the latter.

Moreover, the results recommended that a combinatorial feature should be grouped with at least six features if a couple of features were not sufficient to provide the most optimal prediction performance. There was a dramatic fall in MSE when pairs of a feature were used. The MSE did not always reduce, although features were added sequentially according to the most important to least important sequence. The reason for the MSE not reducing with increasing features might be explained by the dependence of feature(s) at the front of the sequence to generate lower MSE. The dependence is reduced when the number of features is six and above. The recommendation is according to previous studies that performed prediction using different numbers of features, which was either two or greater than five [15, 13, 2, 8].

According to the results in both “Performance of DT with different sizes of feature set” section and “Performance of DT against other classifiers” section, this study discovered that the oDT was highly sensitive and specific in predicting VT/VF in the fourth 5-minute interval, which was 15.1 minutes–20 minutes prior to onset of VT/VF. During the interval, the highest SE and SP of the oDT were observed at 95% and 90%, respectively. The results indicated that a test would be positive 95% if an individual was at risk of imminent VT/VF, and that a test would be negative 90% if an individual was healthy. The oDT outperformed or performed as best as its competitors during the interval. The oDT had much better SE than the fDT, NB, and SVM and higher SP than the SVM, and the second fastest exT.

As have been mentioned, the oDT performed best in the fourth 5-minute interval before the onset of VT/VF. In other intervals, the performance of the oDT was also fairly satisfactory. The oDT could predict VT/VF in any 5-minute interval, although the number of false alerts may be higher in intervals other than the fourth one. This finding did not seem to be consistent with the previous research by Wollmann et al. [7], which stated that abnormality of the mRR could only be found at least 20 minutes before the onset of VT/VF. A possible explanation for this is that may be the study had included multiple features derived from Qon and Soff in addition to R-peaks, which made the abnormality noticeable in an earlier time.

Overall, the oDT had better SE than SP. A small SP loss was still acceptable, because the SE could be a more useful performance indicator than SP in the prediction of imminent VT/VF. An oDT with fairly higher SE but lower SP indicates that the oDT is good for catching upcoming ventricular arrhythmic episodes but may receive a relatively high rate of FP. A higher SE implies that more individuals with risk of VT/VF can be identified correctly, whereas the lower SP means more individuals who are arrhythmia-free had been told of a possibility that VT/VF will happen soon [37]. A higher SE has higher priority than a lower SP, as an individual is better to receive one false alert than miss a warning that could save his life.

Moreover, the oDT took a fairly short time to predict imminent VT/VF, which was 0.64 ms on average. In the real world, 100% accurate prediction is unrealistic, and the oDT cannot predict the exact time of a VT/VF onset. Thus, the shorter the exT of the oDT, the more approaches can be made to avoid or rescue oneself from suffering VT/VF. For instance, a patient may take a deep breath to calm any possible heart rate acceleration or even go the nearest medical center. The prediction result of the oDT may be further investigated to increase the number of correct alerts. Examples of an investigation are blood pressure and photoplethysmography [14].

## Conclusion

This research aimed to propose a fast algorithm for predicting imminent VT/VF while maintaining good SE and SP. At the end of this study, all the objectives had been achieved. This section presents key results that indicate the achievement of objectives.

The first objective of this study was to identify the optimal number of ECG features for prediction of imminent VT/VF, and it was achieved as the “Selection of optimal feature set” section suggested that eight features could be combined for an optimal set. The features were mQRSd, sdSamp, mQamp, mHR, mSamp, mRR, sdQamp, and SDNN. The proposed algorithm with optimal feature set was able to improve 17.95% of exT in exchange for a small increase in MSE (0.0024) and a small decrease in SP (0.45%). Before implementation, this study applied a MATLAB function, Imp, to investigate 12 features derived from QRS complexes. Then, the study selected eight features based on the evaluation results of the effect of features number on SE, SP, and exT of DT. The selection was to avoid redundancy of information for prediction. On the other hand, the purpose of applying the function was to ensure that the eight features were supportive of each other and did not reduce the correct rate of prediction. The optimal set with more than one feature supported the idea raised in other researches [13, 2], which was combinatorial features outperformed single features in prediction.

The second objective of this study was to develop an algorithm for predicting imminent VT/VF. The objective was reached as the “Methods” section demonstrated the development of the algorithm in MATLAB to realize prediction. Before the development, this study first examined essential modules. There were four essential modules, and each module represented different task(s) in the algorithm. The tasks were ECG filtering, fiducial points detection, feature extraction, feature selection, and feature classification. This study applied different methods to the four modules to accomplish the tasks. In the ECG filtering module, this study adapted PT to obtain filtered ECG data and R-peaks of ECG beats. In the fiducial point detection module, the study developed three methods to acquire Qon and Soff of ECG beats correctly. The methods were search windows respective to R-peaks, zero slope detection, and validators. In the feature extraction module, the study used mathematical morphology techniques to compute 12 features that may be associated with VT/VF. In the feature selection module, the study implemented the Imp function and 10-fold CV to choose eight optimal features. Lastly, in the classification module, the study applied the 5-fold CV to train and test three classifiers, which were DT, NB, and SVM. The reasons why these methods were chosen were due to their popularity, short exT or ability to estimate the performance of the proposed algorithm with a limited number of ECG records.

The third objective of this study was to evaluate the performance of the proposed algorithm in five 5-minute intervals, which were 5 minutes, 10 minutes, 15 minutes, 20 minutes, and 25 minutes before initiation of VT/VF. The objective was fulfilled as the “Results” section described the stability of the algorithm in terms of exT but with small fluctuation in terms of SE and SP in all the 5-minute intervals before onset of the arrhythmic event. In this study, high SE was the top priority performance measure, followed by low exT and lastly high SP, because high SE was the most related to high chance of survival. Fluctuation was reflected by the SD of oDT in Table 6, which was 5.05% (SE), 7.52% (SP) and 0.0048 ms (exT). In average, the oDT was a fast classifier (exT = 0.64ms) with good SE (89.28%) and SP (79.33%). In addition, this study recommended that the best timing to predict imminent VT/VF was the 15.1 minutes–20 minutes that preceded the onset. During that time, both the SE and SP of the proposed algorithm succeeded in reaching their peaks at the fourth 5-minute interval, which were 95% and 90% respectively. Performance of the algorithm was evaluated in two situations. The first situation was DT with different feature set (optimal and full feature set), whereas the second one was DT with different classifiers (NB and SVM). From the comparison results, the study found that the exT of oDT was 79.03% faster than NB and its SE was 78.56% higher than SVM, which was a tradeoff for a small SP decrease (not more than 13.54%) compared to its counterparts.

## Supporting information

S1 File Dataset for classification—Training and testing from NSRDB and VFDB. (ZIP)

## References

[1] Zipes DP, Camm aJ, Borggrefe M, Buxton AE, Chaitman B, Fromer M, et al Management of Patients With Ventricular Arrhythmias and the Prevention of Sudden Cardiac Death (Pocket Guideline). 2006;(September):54.

[2] Bayasi N, Tekeste T, Saleh H, Mohammad B, Khandoker A, Ismail M. Low-Power ECG-Based Processor for Predicting Ventricular Arrhythmia. IEEE Transactions on Very Large Scale Integration (VLSI) Systems. 2016;24(5):1962–1974.

[3] Joo SG, Choi KJ, Huh SJ. Prediction of Ventricular Tachycardia by a Neural Network using Parameters of Heart Rate Variability Department of Biomedical Engineering, University of Ulsan College of Medicine, Seoul, Korea Department of Internal Medicine, University of Ulsan Colle. European Heart Journal. 2010;1(c):585–588.

[4] Martin-Yebra A, Demidova M, Platonov P, Laguna P, Martinez JP. Increase of QRS Duration as a Predictor of Impending Ventricular Fibrillation during Coronary Artery Occlusion. In: Computing in Cardiology Conference (CinC), 2013; 2013. p. 133–136.

[5] Rozen G, Kobo R, Beinart R, Feldman S, Sapunar M, Luria D, et al Multipole analysis of heart rate variability as a predictor of imminent ventricular arrhythmias in ICD patients. Pacing and Clinical Electrophysiology. 2013;36(11):1342–1347. 10.1111/pace.12180 23713754

[6] Alemán-Fernández AA, Dorantes-Sánchez M, Castro Hevia J, González LG, Hernández YC, García MAR. Malignant ventricular arrhythmias in patients with implantable cardioverter-defibrillators: electrical signals which are predictors of recurrence. CorSalud (Revista de Enfermedades Cardiovasculares). 2014;6(1):63–69.

[7] Wollmann CG, Gradaus R, Böcker D, Fetsch T, Hintringer F, Hoh G, et al Variations of heart rate variability parameters prior to the onset of ventricular tachyarrhythmia and sinus tachycardia in ICD patients. Results from the heart rate variability analysis with automated ICDs (HAWAI) registry. Physiological measurement. 2015;36(5):1047–61. 10.1088/0967-3334/36/5/1047 25903155

[8] Fairooz T, Khammari H. SVM classification of CWT signal features for predicting sudden cardiac death. Biomedical Physics & Engineering Express. 2016;2:1–10.

[9] Sehamby R, Singh B. Noise cancellation using adaptive filtering in ECG signals: application to biotelemetry. International Journal of Bio-Science and Bio-Technology. 2016;8(2):237–244.

[10] Pan J, Zhu JY, Kee HS, Zhang Q, Lu YQ. A review of compression, ventilation, defibrillation, drug treatment, and targeted temperature management in cardiopulmonary resuscitation. Chinese Medical Journal. 2015;128(4):550–554. 10.4103/0366-6999.151115 25673462 PMC4836263

[11] Tereshchenko LG, Fetics BJ, Domitrovich PP, Lindsay BD, Berger RD. Prediction of Ventricular Tachyarrhythmias by Intracardiac Repolarization Variability Analysis. Circulation-Arrhythmia and Electrophysiology. 2009;2(3):276–284. 10.1161/CIRCEP.108.829440 19808478

[12] Ragupathi L, Pavri BB. Tools for risk stratification of sudden cardiac death: A review of the literature in different patient populations. Indian Heart Journal. 2014;66(SUPPL. 1):S71–S81. 10.1016/j.ihj.2013.12.035 24568833 PMC4237289

[13] Riasi A, Mohebbi M. Prediction Of Ventricular Tachycardia Using Morphological Features Of ECG Signal. 2015 The International Symposium on Artificial Intelligence and Signal Processing (AISP). 2015; p. 170–175.

[14] Lee HJ, Shin SY, Seo MS, Nam GB, Joo SG. Prediction of Ventricular Tachycardia One Hour before Occurrence Using Artificial Neural Networks. Nature Publishing Group. 2016;(April):1–7. 10.1038/srep32390 PMC4999952 27561321

[15] Ebrahimzadeh E, Pooyan M, Bijar A. A Novel Approach to Predict Sudden Cardiac Death (SCD) Using Nonlinear and Time-Frequency Analyses from HRV Signals. Plos One. 2014;9(2):1–14. 10.1371/journal.pone.0081896 PMC3913584 24504331

[16] Murugappan R. *A death too soon*; 2013. [Newspaper] The Star, 18 August 2013.

[17] Karimuribo ED, Mutagahywa E, Sindato C, Mboera L, Mwabukusi M, Kariuki Njenga M, et al A Smartphone App (AfyaData) for Innovative One Health Disease Surveillance from Community to National Levels in Africa: Intervention in Disease Surveillance. JMIR Public Health and Surveillance. 2017;3(4):e94 10.2196/publichealth.7373 29254916 PMC5748470

[18] Colunas, M F M and Fernandes, J M A and Oliveira, I C and Cunha, J P S. Droid Jacket: Using an Android based smartphone for team monitoring. In: Wireless Communications and Mobile Computing Conference (IWCMC), 2011 7th International; p. 2157–2161.

[19] Watanabe H, Kawarasaki M, Sato A, Yoshida K. Development of wearable heart disease monitoring and alerting system associated with smartphone. In: e-Health Networking, Applications and Services (Healthcom), 2012 IEEE 14th International Conference on, 2012; p. 292–297.

[20] Sohn K, Dalvin SP, Merchant FM, Kulkarni K, Sana F, Abohashem S, et al Utility of a Smartphone Based System (cvrPhone) to Predict Short-term Arrhythmia Susceptibility. Scientific Reports. 2019;9(1):14497 10.1038/s41598-019-50487-4 31601824 PMC6787075

[21] Lee H, Shin SY, Seo M, Nam GB, Joo S. Prediction of Ventricular Tachycardia One Hour before Occurrence Using Artificial Neural Networks. Scientific Reports. 2016;6(1):32390 10.1038/srep32390 27561321 PMC4999952

[22] Goldberger AL, Amaral LAN, Glass L, Hausdorff JM, Ivanov PC, Mark RG, et al Physiobank, physiotoolkit, and physionet components of a new research resource for complex physiologic signals. Circulation. 2000;101(23):e215–e220. 10.1161/01.cir.101.23.e215 10851218

[23] Sedghamiz H. Matlab Implementation of Pan Tompkins ECG QRS detector; 2014. Available from: https://www.researchgate.net/publication/313673153_Matlab_Implementation_of_Pan_Tompkins_ECG_QRS_detector

[24] Zhao QB, Zhang LQ; IEEE. ECG feature extraction and classification using wavelet transform and support vector machines. 2005;2:1089–1092.

[25] Yan H, Jiang Y, Zheng J, Peng C, Li Q. A multilayer perceptron-based medical decision support system for heart disease diagnosis. Expert Systems with Applications. 2006;30(2):272–281.

[26] Jen K, Hwang Y. ECG feature extraction and classification using cepstrum and neural networks. Journal of Medical and Biological Engineering. 2008;28(1):31–37.

[27] Mandala S, Tham CD. ECG Parameters for Malignant Ventricular Arrhythmias: A Comprehensive Review. Journal of Medical and Biological Engineering. 2017;37(4):441–453. 10.1007/s40846-017-0281-x 28867990 PMC5562779

[28] Rizal A, Lestari P, Mandala S, Satrijo B, Widito S. Neural Network based—Arrhythmia Monitoring Device: A Pivotal Clinical Trial. International Journal of Innovative Technology and Exploring Engineering. 2020;9(3S):346–349.

[29] Raeiatibanadkooki M, Quachani SR, Khalilzade M, Bahaadinbeigy K. Real time processing and transferring ECG signal by a mobile phone. Acta Informatica Medica. 2014;22(6):389–392. 10.5455/aim.2014.22.389-392 25684847 PMC4315648

[30] The MathWorks, Inc. MATLAB R2015a documentation; 2015. [Software Manual].

[31] Siroky DS. Navigating Random Forests and related advances in algorithmic modeling. Statistics Surveys. 2009;3(0):147–163.

[32] James G, Witten D, Hastie T, Tibshirani R. An Introduction to Statistical Learning In: Springer Texts in Statistics. vol. 7; 2000 p. 175–197.

[33] Refaeilzadeh P, Tang L, Liu H. On Comparison of Feature Selection Algorithms. In: Proceedings of AAAI workshop on evaluation methods for machine learning II. vol. 3; 2007.

[34] Mudry A, Tjellström A. Historical background of bone conduction hearing devices and bone conduction hearing aids. Advances in Oto-Rhino-Laryngology. 2011;71:1–9. 10.1159/000323569 21389699

[35] American Heart Association Inc, European Society of Cardiology. Guidelines Heart rate variability. European Heart Journal. 1996;17:354–381. 8737210

[36] Sachdev M, Fetics BJ, Lai S, Dalal D, Insel J, Berger RD. Failure in short-term prediction of ventricular tachycardia and ventricular fibrillation from continuous electrocardiogram in intensive care unit patients. Journal of Electrocardiology. 2010;43(5):400–407. 10.1016/j.jelectrocard.2010.02.005 20378124 PMC2914842

[37] Lalkhen AG, McCluskey A. Clinical tests: sensitivity and specificity. Continuing Education in Anaesthesia Critical Care & Pain. 2008;8(6):221–223.

